# Microsphere Polymers in Molecular Imprinting: Current and Future Perspectives

**DOI:** 10.3390/molecules25143256

**Published:** 2020-07-17

**Authors:** Tirza Ecclesia Orowitz, Patria Pari Agnes Ago Ana Sombo, Driyanti Rahayu, Aliya Nur Hasanah

**Affiliations:** Department of Pharmaceutical Analysis and Medicinal Chemistry, Faculty of Pharmacy, Universitas Padjadjaran, Jl. Raya Bandung Sumedang KM 21.5, Sumedang 45363, Indonesia; tirza17001@mail.unpad.ac.id (T.E.O.); patria17001@mail.unpad.ac.id (P.P.A.A.A.S.); driyanti.rahayu@unpad.ac.id (D.R.)

**Keywords:** molecularly imprinted microsphere, microsphere polymer, precipitation polymerisation, controlled/‘living’ radical precipitation polymerisation, pickering emulsion polymerisation, suspension polymerisation

## Abstract

Molecularly imprinted polymers (MIPs) are specific crosslinked polymers that exhibit binding sites for template molecules. MIPs have been developed in various application areas of biology and chemistry; however, MIPs have some problems, including an irregular material shape. In recent years, studies have been conducted to overcome this drawback, with the synthesis of uniform microsphere MIPs or molecularly imprinted microspheres (MIMs). The polymer microsphere is limited to a minimum size of 5 nm and a molecular weight of 10,000 Da. This review describes the methods used to produce MIMs, such as precipitation polymerisation, controlled/‘Living’ radical precipitation polymerisation (CRPP), Pickering emulsion polymerisation and suspension polymerisation. In addition, some green chemistry aspects and future perspectives will also be given.

## 1. Introduction

Molecularly imprinted polymers (MIPs) are specific crosslinked polymers that exhibit binding sites for template molecules. MIPs are widely used because of their high selectivity and affinity for template molecules [1,2]. MIPs have good thermal and chemical stability under high or low pH and temperature [3]. MIPs have advantages over their biological counterparts as they are inexpensive, simple, stable, reproducible, induce strong chemical bonds, and demonstrate stability to heat and pressure [4]. MIP formation requires monomers, molecular templates, initiators, crosslinkers and solvents [5,6]. The complex between the monomer and the template molecule requires either non-covalent interactions or covalent interactions [7]. In recent studies, organic solvents have shown better specific binding ability than aqueous solvents [8].

MIPs have been developed in various application areas of biology and chemistry, such as in selective separation and sample preparation [9,10,11,12], nanocomposite materials [13], biological assays [14,15,16], catalysis [17], drug delivery [18,19], sensors [20,21,22,23], bioimaging and optical devices [24], food safety and environmental applications [25,26], enantioseparation [27,28], natural product extraction [29,30] and rare element purification [31]. MIPs can be obtained by various methods, including bulk polymerisation, precipitation polymerisation [32,33], suspension polymerisation, emulsion polymerisation, two-step swelling polymerisation [34], electropolymerisation [35], electrospinning [36], sol-gel imprinting [37] and phase inversion [38]. However, according to Bai et al. [39], these methods have some problems, such as an irregular material shape (which is not ideal for chromatographic purposes [40,41] when used as the stationary phase, causing irreproducible results and asymmetrical peaks), low affinity, weak binding forces and incomplete removal of template molecules, which limits extraction efficiency and imprinting ability. This review provides recent and future perspectives of microsphere polymers in molecular imprinting, including new polymer synthesis routes that overcome the drawbacks of conventional methods.

## 2. Microsphere Polymers

Microspheres are microscopic-sized spherical particles in the range of 1–1000 µm in diameter, although particles over 1000 µm in size have also been described as microspheres. Microspheres have a variety of functions, especially in the biological, clinical and chemical fields. Polymer microspheres have been developed for controlled drug delivery [42,43] and used in solid-phase extraction. This type of polymer can also be used as a diagnostic method for diseases associated with the blood [43].

Microspheres must possess specific characteristics of size, surface area, diffusion ability, stability, biocompatibility and safety. The polymer microsphere is limited to a minimum size of 5 nm and a molecular weight of 10,000 Da. The surface area of 1 g of microspheres with a diameter of 0.1 μm is 60 m^2^. The surface area will affect the chemical reactions that occur on the microsphere surface [43,44]. Molecularly imprinted microspheres (MIMs) are used in a number of applications, such as competitive ligand bond tests, solid-phase molecular extraction (SPME), microsphere sensors and capillary electrochromatography [40]. Other applications have used MIMs for sustained drug release [45,46,47]. Microsphere polymers prepared using the molecular imprinting method show a more uniform pore size and higher specificity for the template molecule compared to other methods. However, the absorption capacity of the microsphere polymer is higher than that of MIMs [48]. Table 1 shows other applications of MIMs with different templates, monomers, crosslinkers and polymerisation techniques.

## 3. Preparation of Molecularly Imprinted Microspheres

### 3.1. Precipitation Polymerisation

Precipitation polymerisation is one of the polymerisation methods that are widely used in the preparation of MIMs. Ye et al. [40,41] stated that uniform MIP microspheres could be produced using precipitation polymerisation under a very dilute conditions, but this requires a greater amount of solvent than that used in the conventional method (approximately <5% [6] of the total volume of monomer loading relative to the solvent), producing an average particle diameter around 0.2–0.3 μm.

**Figure 1:**
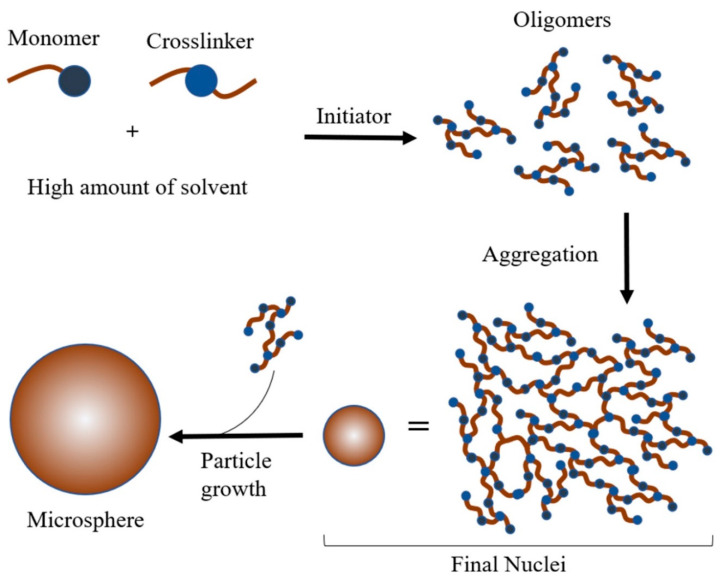
The mechanism of precipitation polymerisation.

The mechanism of precipitation polymerisation was proposed by Li and Stöver [121], where initiation takes place in a homogenous solution containing monomers, crosslinkers, initiators and solvent. In conventional precipitation polymerisation, polymer formation starts by forming nuclei and oligomers. As these oligomers grow, they will begin to crosslink. Instead of overlapping with one another, the polymer chains grow individually by adding newly formed oligomers and monomers from the solvent. The chain length grows until it exceeds its solubility and eventually precipitates from the solution with uniformly spherical morphologies [41] (Figure 1). As reported previously [40], conventional MIPs have an irregular shape and size, while MIMs produce spherical microgels with a narrow size distribution. The formation of MIMs is dependent on various factors. For example, to produce larger particles, adjusting the solubility parameter of the growing polymer is critical, notably when the goal is simultaneously the control of polymer morphology. The monomer concentration, solvent mixture and method of agitation also play roles in the development of MIMs [6,51].

Polymerisation of divinylbenzene (DVB) or trimethylolpropane trimethacrylate (TRIM) as the crosslinker in acetonitrile together with azobisisobutyronitrile (AIBN, initiator) is known as the basic procedure of precipitation polymerisation, and thus has been widely studied with various templates [40,41,70,121]. Polymerisation is induced by either thermal or UV irradiation. The microspheres can be readily obtained through centrifugation [40]. In precipitation polymerisation, the morphology of the polymer is not significantly affected by the presence or absence of a template [6,52,122], but this has an impact on particle growth [70].

As mentioned above, in this case, the precipitation of the chains is caused by crosslinking. This approach requires no stabiliser or surfactant, which is beneficial in molecular imprinting, since these compounds can disrupt template-monomer complex formation. However, the fact that microsphere formation occurs in very dilute solutions (using solvent >95 wt.%) makes this technique economically unfavourable and leads to environmental pollution [51,52]. This problem led Jin et al. [52] to develop modified precipitation polymerisation (MPP), which needs a smaller amount of solvent (about 50 wt.%). This method uses a mixture of toluene and alkane (mineral oil) instead of the normal non-proton solvents (e.g., acetonitrile, chloroform, toluene). The morphology of particles was controlled by the volume ratio of mineral oil to toluene. MIMs obtained via conventional precipitation polymerisation and MPP had similar selectivity, in terms of imprinting and separation factors.

However, the concentration of the crosslinker affects the binding capacity and selectivity. Some researchers [88,109,121] have stated that MIMs prepared using the minimum concentration of crosslinker have the highest binding capacity and selectivity, but excess could reduce stability due to the increased solvency of the continuous phase and particle swelling. A study conducted by Jiang et al. [88] showed that agitation has an influence on MIM production by MPP. MIMs were achieved only without agitation, which is different from earlier precipitation polymerisation methods in which MIMs could be produced with or without agitation. In MPP, the coagulation level increased along with an increase in the speed of agitation, as increased collision frequencies among the particles allowed them to coagulate.

However, another hurdle that precipitation polymerisation has to face is the low rate of the reaction as a result of using a low monomer concentration, making the polymerisation process relatively slow. Furthermore, a large amount of solvent might adversely impact the template–monomer complex, shifting the equilibrium to the uncomplexed form and decreasing the amount of imprinted binding sites. To overcome this, some studies [51,53,54] have successfully prepared MIMs by modifying the conventional precipitation polymerisation method, so that very high monomer concentrations could be added (≥25–40 *v*/*v*%), by using appropriate solvent or solvent mixtures. The solvent needs to be thermodynamically poor, i.e., it either has a large molar volume or has a significantly different solubility parameter compared to the polymer. It may be a problem to dissolve the monomer, but this can be resolved by using the right co-solvents. Therefore, the polymer will be swollen mainly in the co-solvent, while the incompatible solvent is expected to segregate the growing particles. The use of a good co-solvent results in smoothly dispersed microspheres, while segmented microparticles are obtained when using poorer co-solvents [51].

Recent studies showed that the preparation of MIMs using precipitation polymerisation has successfully led to different templates [55,56,58,59,60,61,90,91,92,93,94] and sensors [107], and extracted various analytes from natural ingredients [95,111,112,113], biological samples [62,63,64,65,108], foods [96,97,109,114,115] and wastewater [98,99]. As an easy, simple and commonly used strategy for producing MIMs, precipitation polymerisation has great potential for developing advanced strategies in combination with other approaches (molecularly imprinted solid phase extraction (MISPE), biochemical sensors, high performance liquid chromatography (HPLC), etc.) (see Table 1).

### 3.2. Controlled/‘Living’ Radical Precipitation Polymerisation (CRPP)

As mentioned above, precipitation polymerisation has proven versatile for preparing MIMs, since it does not require a stabiliser or surfactant and is easy to operate. Most precipitation polymerisation reactions involve free radical polymerisation, which is slow to initiate but has quick chain propagation. However, the rate cannot be controlled, and it is susceptible to chain transfer and termination, leading to a broad size distribution of the polymer [69]. More advanced polymerisation techniques, such as controlled/‘living’ radical polymerisation (CRP), have drawn interest for producing MIMs due the improved control regarding composition, molecular weight and end group functionality [57]. The principle of CRP lies in the equilibrium of growing free radicals or active species and dormant species, which can minimise chain breaking reactions and the instantaneous initiation of all chains [123,124]. The CRP methods that are considered most efficient are atom transfer radical polymerisation (ATRP), stable free radical polymerisation (SFRP), nitroxide mediated polymerisation (NMP) and reversible addition-fragmentation chain transfer (RAFT).

The presence of CRP-’living’ groups on the polymer surface are important since the ‘living’ groups are useful for advanced surface modification (e.g., surface modification by the incorporation of a hydrophilic comonomer). However, the MIMs obtained via a precipitation polymerisation mechanism based on traditional free radical polymerisation commonly do not possess ‘living’ groups on their surfaces. As a developing polymerisation technique, controlled/‘living’ radical precipitation polymerisation (CRPP) occurs by the incorporation of the CRP mechanism into the precipitation polymerisation system, thus merging the superiority of CRP with a traditional precipitation polymerisation system. Several CRPP methods that have been developed will be discussed below.

#### 3.2.1. Atom Transfer Radical Precipitation Polymerisation (ATRPP)

**Figure 2:**
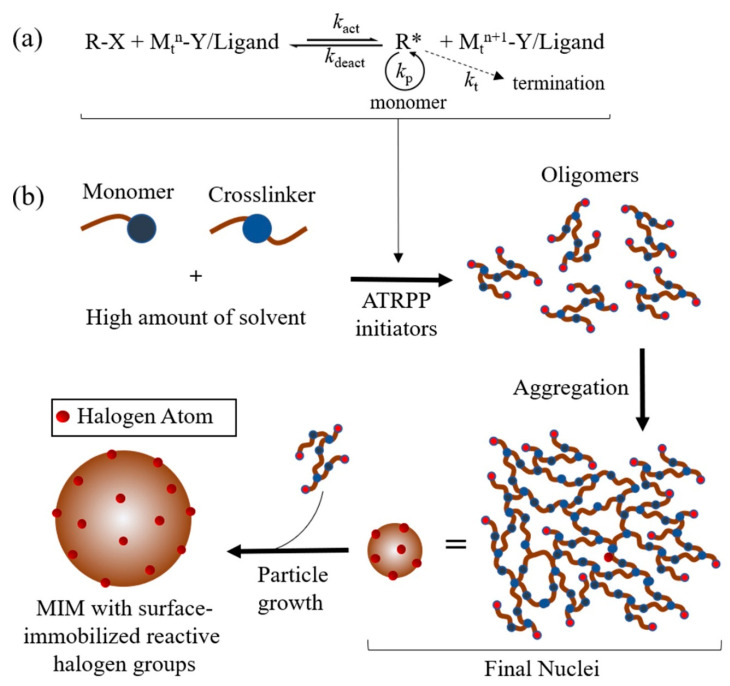
The mechanism of atom transfer radical polymerisation (ATRP) (**a**) and ATRPP (**b**). R-X, dormant species; M_t_^n^, transition metal in its lower oxidation state; M_t_^n+1^, transition metal in its higher oxidation state; Y, may be another ligand or the counterion; M_t_^n^-Y/Ligand, transition metal complex; *k*_act_, rate constant of activation; *k*_deact_, rate constant of deactivation; *k*_p_, rate constant of propagation; *k*_t_, rate constant of termination; ATRPP initiators, alkyl halide/CuCl/Ligand in normal ATRP or AIBN/CuCl_2_/Ligand in reverse ATRP.

Since 1995, the atom transfer radical polymerisation (ATRP) approach has drawn considerable interest because of its versatility in polymer synthesis with specific functionalities and a wide scope of monomer, initiator and catalyst utilisation. The name ATRP stands for the atom transfer process, which is the main element that mediates the uniform growth of polymer chains. The reaction is based on a rapid and dynamic equilibrium among the active species (radicals) and dormant species (alkyl halides) with transition metal complexes that act as reversible halogen atom transfer reagents (Figure 2a). This system carries a very minimal radical concentration. ATRPP processes have a rate constant of activation (*k*_act_) and deactivation (*k*_deact_). Polymer chains mainly grow by capturing intermediate radicals to monomers with the rate constant of propagation (*k*_p_). Termination with the rate constant of termination (*k*_t_) additionally occurs, commonly through radical coupling and disproportionation; however, in well-controlled ATRP, less than 5% of growing polymer chains undergo termination. Initiators determine the end groups of polymers obtained by ATRP. For example, when the initiator is an alkyl halide (or arenesulphonyl halide), one end group of the polymer will be halide and the other end will be an alkyl (or arenesulphonyl) group. This characteristic is highly advantageous for further polymer surface modification [1,125].

Two types of ATRP can be observed based on the initiation system, namely normal and reverse ATRP. In normal ATRP, initiator radicals originate from the reaction between alkyl halide and transition metal complexes in a lower oxidation state (e.g., Cu(I)/Ligand). As for reverse ATRP, initiator radicals originate from a conventional initiator (e.g., AIBN) at the onset of polymerisation, which are then deactivated by a transition-metal complex in its higher oxidation state (e.g., Cu(II)/ligand). The equilibrium for the active species (radicals) and dormant species (alkyl halide) of these types of system can be achieved rapidly at the beginning of the polymerisation process [125].

Atom transfer radical precipitation polymerisation (ATRPP) is the incorporation of the ATRP system with a precipitation polymerisation procedure, which is simply a substitute for the conventional initiator (e.g., AIBN) with an ATRP initiator, such as the initiator in normal or reverse ATRPP (Figure 2b). Similarly to conventional precipitation polymerisation, the processes of nucleation and particle growth also occur in ATRPP. The difference between ATRPP and traditional radical precipitation polymerisation (TRPP) is, in ATRPP, all the ATRP initiators should be immediately converted into macroinitiators as soon as the process begins, which implies that the total yield of the newly formed polymer chains throughout the particle growth stage ought to be unimportant. Conversely, in TRPP, new oligomers are continuously produced during the process. In this manner, the mechanism of particle growth in ATRPP ought to be different from TRPP, as the polymer in ATRPP grows by catching monomers directly from the solution via the process of surface-initiated controlled polymerisation. This shows that the controlled attributes of ATRPP have major roles in uniform particle growth and size [126]. Furthermore, the presence of a surface-immobilised halogen group in the obtained MIMs facilitates further surface modification. The ability to modify the surface of MIMs to achieve better compatibility in various solvent systems has made them immensely applicable in different fields.

Zu et al. [1] proposed the incorporation of the ATRP system into precipitation polymerisation to form MIMs with a surface consisting of reactive halogen groups. This study demonstrated the use of 4-vinylpyridine (4-VP) as the monovinyl functional monomer, ethylene glycol dimethacrylate (EGDMA) as the crosslinking monomer, ethyl 2-chloro-propionate as the initiator, CuCl as the transition metal salt, *N,N,N′N′,N′*-pentamethyldiethylenetriamine (PMDETA) as the ligand and acetonitrile divinyl as the solvent for ATRPP and compared it with TRPP using 4-VP, EGDMA, AIBN and acetonitrile. The processes were executed in acetonitrile (97% of the total volume) at 60 °C.

The diameters of the MIMs produced via TRPP were 10 times smaller than those produced via both normal and reverse ATRPP (2–5 µm vs. 200–430 nm), implying that the combined system in ATRPP significantly affects the particle size of the obtained MIMs. This relatively larger MIMs were useful in diverse applications, for example in HPLC as the stationary phase. Furthermore, the equilibrium loading capacities of the MIMs obtained by normal and reverse ATRPP systems were identical, yet higher than the MIMs obtained by TRPP. Another difference was shown regarding high-affinity site densities in which MIMs prepared by ATRPP had significantly higher high-affinity site densities on their surfaces compared to those obtained by TRPP [1].

Jiang et al. [126] investigated some polymerisation parameters in ATRPP systems that could impact the morphologies and yields of the MIMs. The outcomes indicated that the MIM particles prepared without magnetic stirring were relatively polydisperse, while monodisperse MIMs were acquired by applying suitable stirring rates (around 90–180 rpm) under similar conditions. As for the effect of monomer loading, both the yields and sizes of the MIMs increased along with increasing the monomer loading, similarly as observed in TRPP. The polymerisation rates were additionally found to increase with increasing both the initiator and catalyst concentrations, which is reasonable in such a highly diluted polymerisation system.

To demonstrate its general applicability, Jiang et al. [126] also performed ATRPP with the use of hydrophilic functional monomers (i.e., 2-hydroxyethyl methacrylate (HEMA) and acrylamide (AAm)) to produce highly crosslinked copolymer microspheres. The ‘livingness’ of the MIMs was also shown by the ability to perform surface modification with hydrophilic functional monomers (i.e., *N*-isopropylacrylamide (NIPAAm) and HEMA) via the surface-initiated ATRP, producing polymer brush-grafted MIMs with significantly improved water dispersion stability and surface hydrophilicity.

To create a more versatile methodology of ATRPP that can perform under mild reaction conditions, Jiang et al. [127] conducted the first ambient temperate MIM synthesis without any external initiators, which is of great environmental and commercial significance and highly applicable for monomers that are sensitive to high temperatures. Ambient temperature ATRPP produced MIMs with smaller particle sizes compared to MIMs obtained via ATRPP at 60 °C. This phenomenon might be due to the decrease in solubility of the oligomers along with decreasing temperature (i.e., the relatively lower molecular weight oligomers became insoluble at ambient temperature) or as a result of the decreased polymerisation rates at lower temperatures. ATRPP performed at an ambient temperature is an efficient methodology to synthesise various MIMs with diverse potential uses that are cost-effective and environmentally friendly.

Among various stimuli-responsive MIPs that have been developed, photoresponsive MIMs have attracted particular attention, since light is a stimulus that can be introduced immediately and conveyed with high accuracy in exact amounts [128]. Fang et al. [100] described the first method to achieve MIMs containing an azo group with stimuli-responsive properties, both thermo- and photoresponsive, in aqueous media by utilising an acetonitrile-soluble azo functional monomer with a pyridine group (i.e., 4-((4-methacryloyloxy)phenylazo)pyridine [MAzoPy]), a crosslinker, a template and polyNIPAAm (PNIPAAm) brushes for surface grafting via surface-initiated ATRPP. The photoresponsive properties were exhibited by equilibrium binding experiments, where the grafted azo-containing MIMs were obtained in pure water drops after exposure to UV light (365 nm), showing that exposure to UV light could lead to an alteration of the spatial arrangement of MIM binding properties, resulting in changes in their affinity. This was further demonstrated by their photoregulated properties that can take up and release the template in a pure aqueous environment, with repeated photoswitching cycles. This method is an excellent way of developing advanced intelligent MIMs with both water-compatible and stimuli-responsive (thermo- and photoresponsive) binding properties for various templates. Additionally, the azo-containing MIMs with surface-grafted PNIPAAm brushes have a great potential in the future for extraction, smart separation, assays and intelligent drug delivery.

Other researchers have developed ATRPP for the separation of sulphamethazine [66] and 17β-oestradiol [49] (see Table 1). A new ATRPP method could be conducted in the future, for example using the activators regenerated by electron transfer (ARGET)-ATRP approach [129].

#### 3.2.2. Iniferter-induced ‘Living’ Radical Precipitation Polymerisation (ILRPP)

**Figure 3:**
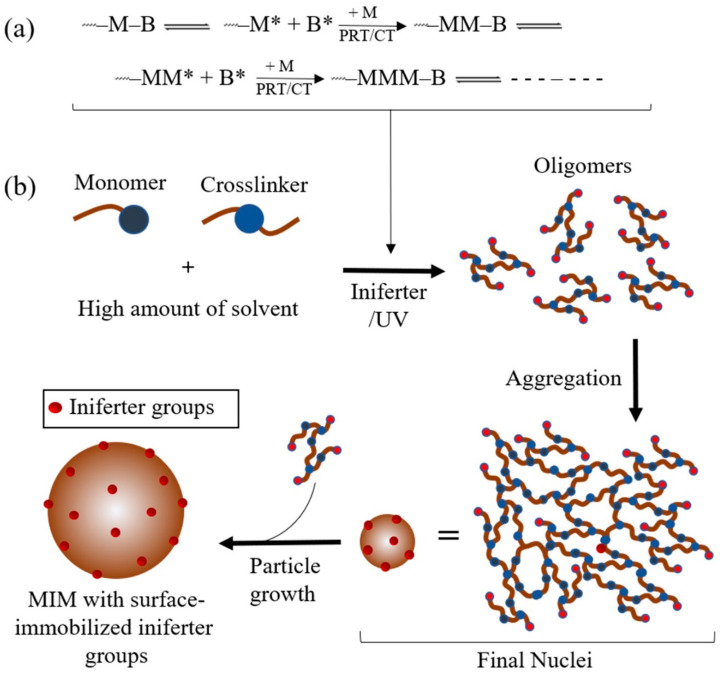
The mechanism of iniferter-induced ‘living’ radical polymerisation (ILRP) (**a**) and ILRPP (**b**). M, monomer; B, small radical; M*, propagating radical; B*, less reactive or nonreactive radical; PRT, primer radical termination; CT, chain transfer.

Since 1982 [130], iniferter-induced ‘living’ radical polymerisation (ILRP) has drawn wide interest for its versatility in controlled macromolecular structure design. The controllable properties of ILRP are based on the use of an iniferter agent (initiator-transfer agent-terminator, generally dithiocarbamates), which can act as either a dormant species (iniferter) or an active species (propagating radical) with a non-reactive radical (dithiocarbamate radical) under suitable conditions (the latter acts as a capping agent for the propagating radicals) (Figure 3a). The obtained MIMs are generally end-capped with an iniferter group, making them able to produce block polymers by iniferter-induced chain-extension polymerisation [101,131].

Iniferter-induced ‘living’ radical precipitation polymerisation (ILRPP) is the most recently developed CRPP by incorporating the ILRP system into precipitation polymerisation, which simply substitutes the conventional initiator (e.g., AIBN) with an iniferter agent; an iniferter is a compound that acts as an initiator, transfer agent and terminator at the same time during radical polymerisation [130] (Figure 3b). Being similar to conventional precipitation polymerisation, the processes of nucleation and particle growth also occur in ILRPP. The mechanism of particle formation in ILRPP is similar to that in ATRPP, where every part of the iniferters in ILRPP is immediately converted into macroiniferters at the onset of polymerisation, which implies that the total yield of newly formed polymer chains throughout the particle growth stage ought to be unimportant. Like ATRPP, the polymer particles in the ILRPP grow by catching monomers straight from the solution via the process of surface-initiated controlled polymerisation. This shows that the controlled attributes of ATRPP have an impact on uniform particle growth and size. Moreover, the existence of ‘living’ iniferter groups on MIM surfaces facilitates further surface modification [132].

Li et al. [101] first demonstrated ILRPP by using EGDMA as the functional monomer and benzyl dithiocarbamate (BDC) as the iniferter agent in acetonitrile as the solvent. With this photoiniferter, UV light irradiation acts as the initiator of polymerisation at 37 °C (ambient temperature). Photo-ILRPP shows significant superiority among other thermal precipitation polymerisation reactions, as it can be conducted at relatively low temperatures that are suitable for the complexation of template-functional monomers.

The factors that can affect ILRPP have also been studied [101]. As both the temperature and iniferter concentration increase, the polymerisation rate also increases, indicated by an increased polymer yield. However, an increase in the iniferter concentration also causes an increase in MIM particle size, and an increase in polymerisation temperature causes a decrease in particle size. This might be because an increase in the initiator concentration can increase the instantaneous concentration of the growing oligomeric radicals and lead to a larger final particle, while increasing the reaction temperature improves the solubility of the oligomer chains but also increases the decomposition rate of the initiator. Therefore, the particle size is inversely proportional to the reaction temperature, as smaller MIMs are formed at higher temperatures and larger ones are produced at lower temperatures [133]. It can be concluded that the particle sizes that are obtained via ILRPP can be efficiently controlled by adjusting the reaction conditions.

Li et al. [101] also demonstrated the surface modification of MIMs via surface-initiated ILRPP by using NIPAAm. The incorporation of PNIPAAm onto the MIMs was observed by the increased weight and diameter of grafted polymer particles. The enhanced water dispersion stability of grafted MIMs was also shown in this study.

To demonstrate the wide application of ambient temperature ILRPP, another study [132] used poly(4-VP-co-EGDMA) and poly(GMA-co-EGDMA) (GMA; glycidyl methacrylate) to synthesise MIMs. This study also demonstrated the ‘livingness’ of the MIMs using hydrophilic functional monomers (i.e., NIPAAm and HEMA) via surface-initiated ILRPP. The increase in diameter and weight of grafted MIMs compared with the ungrafted ones showed that functional polymer brushes had been grafted onto the MIMs. The MIM particles grafted with PNIPAAm and PHEMA brushes also showed a significant increase in dispersion stability in water and a decrease in static water contact angles. The ILRPP technique performed at an ambient temperature is a general and efficient methodology to synthesise various MIMs for diverse potential fields that are cost-effective and environmentally friendly.

Other studies have performed ILRPP with different templates, including glutathione [67] and thymopentin (TP5) [68] (see Table 1). In contrast with ATRPP, which has limited applications due to the use of large amounts of acidic functional monomers or templates that could possibly deactivate the metal catalyst, the ILRPP methodology is compatible with many molecular imprinting systems, giving it tremendous potential for the future preparation of MIMs for various applications, such as drug delivery, chemical sensors and bioanalytical applications.

#### 3.2.3. Reversible Addition-Fragmentation Chain Transfer Precipitation Polymerisation (RAFTPP)

**Figure 4:**
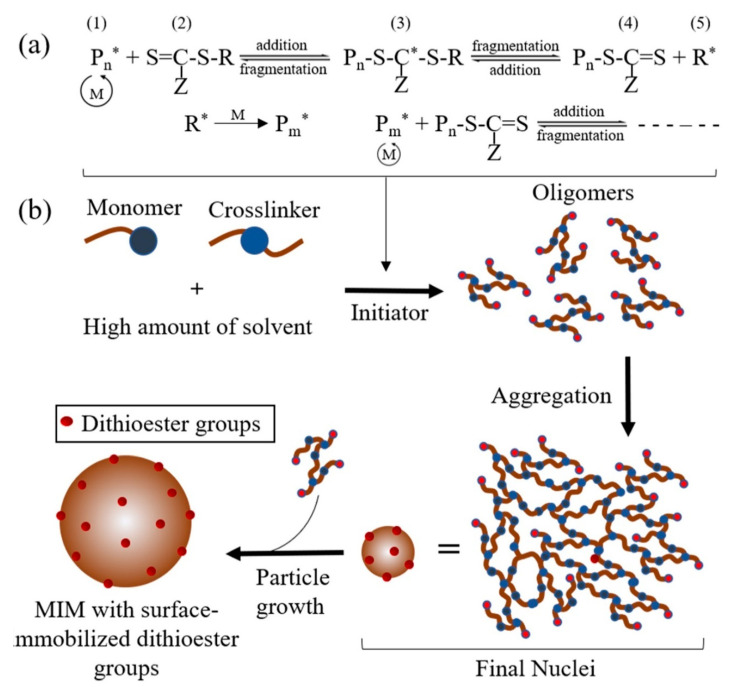
The mechanism of the reversible addition-fragmentation chain transfer (RAFT) polymerisation (**a**), RAFTPP (**b**). M, monomer; (1), propagating radical; (2), thiocarbonylthio compound as RAFT agent; S, sulphur; R, free radical leaving-group that is capable of reinitiating polymerisation; Z, group that modifies the activity of the RAFT agent; (3), intermediate radical; (4), dormant species; (5), fragment radical.

Among all the CRPs that have been developed, reversible addition-fragmentation chain transfer (RAFT) polymerisation has become one of the most widely used CRP methods because of its effectiveness, applicability with a very broad range of monomers and its ability to perform at a mild reaction temperature [134]. The principle of RAFT polymerisation involves an appropriate chain transfer agent (CTA or RAFT agent, usually a thiocarboylthio compound such as dithioesters, trithiocarbonates, xanthates, dithiocarbamates, etc.), resulting in a product of chain transfer that also acts as a chain transfer agent with identical characteristics to its precursor transfer agent but differing in molecular weight (degenerate or degenerative chain transfer) [135]. This process also results in fast equilibrium among the active species (propagating radicals) and dormant species (thiocarbonylthio-terminated chains), creating an equal probability of forming narrowly polydisperse polymers (Figure 4a). When the process is done, most of chains will maintain the thiocarboulthio end group so it can be isolated as a stable material [5].

First introduced by Pan et al. [5], reversible addition-fragmentation chain transfer precipitation polymerisation (RAFTPP) has been widely used by combining RAFT with precipitation polymerisation in MIM preparation. Merging RAFT polymerisation with precipitation polymerisation is as simple as introducing a RAFT agent into the precipitation polymerisation, producing a MIM with reactive thiocarboylthio groups on its surface, thus creating favourable properties for further surface modification (Figure 4b).

Pan et al. [5] demonstrated RAFTPP using 2,4-dichlorophenoxyacetic acid (2,4-D as the template with 4-VP as the functional monomer, EGDMA as the crosslinker, AIBN as the initiator, a methanol and water mixture (4:1 in volume, ≥98% of the total reaction volumes) as the solvent and cumyl dithiobenzoate (CDB) as the CTA and compared it with TRPP under similar conditions without adding CDB. All reactions were performed at 60 °C for 24 h. The template was then removed from the MIPs via centrifugation. In the end, white MIP or non-molecularly imprinted polymer (NIP, a MIP without a template) particles were obtained via TRPP, while RAFTPP produced light pink MIP/NIP particles, demonstrating the successful incorporation of dithioester groups into the system.

TRPP produced only irregular MIPs, while uniform non-molecularly imprinted microspheres (NIMs) were produced under similar conditions. This result suggests that combining RAFT into precipitation polymerisation might have a significant impact on the morphology and particle sizes of MIM/NIM. RAFTPP produced MIM with enhanced binding capacity, selectivity for a specific target, a high binding constant, possible maximum number for high-affinity sites and a greater high-affinity binding site density that those prepared by TRPP [5]. Since then, researchers have developed RAFTPP into various templates [57,69,116]. However, these MIPs are generally only compatible with organic solvents and predominantly fail to undergo specific binding in a pure aqueous environment, thus limiting their application in various fields.

In response to this drawback, some studies [8,71,72,102,103,105,106] developed an advanced approach that effectively formed a pure water-compatible MIM via surface-initiated RAFTPP. The procedure differed in the use of a hydrophilic comonomer (such as HEMA, *N*-isopropylacrylamide [NIPAAm], acrylic acid, azobenzene [azo]) as the grafted polymer. According to Pan et al. [102], MIM/NIM particles grafted with polyNIPAAm (PNIPAAm) brushes showed improved dispersion stability in a pure water environment at ambient temperature, making it not only pure water-compatible, but also stimuli-responsive (mimicking biological receptors, and highly responsive to external stimuli such as temperature). The properties of the hydrophilic surface in the grafted and ungrafted MIM/NIM particles could be investigated by comparing their static water contact angle. This outcome showed a significant difference between the grafted and ungrafted MIM film, as the grafted MIM film showed greater hydrophilicity compared to the ungrafted one. The grafted MIM/NIMs also showed a reduction in non-specific binding in pure aqueous solution, thus increasing water compatibility. This approach was highly useful, since it allows any kind of hydrophilic monomer to be grafted onto the MIM surface and requires no additional of functional or hydrophilic comonomers in the molecular imprinting system, thus making it less complicated and more efficient (requires no time for the optimisation of MIM formulation components). The stimuli-responsive MIMs (in this case, thermo-responsive) showed significantly decreased specific template binding of the grafted MIMs at a higher temperature (45 °C) compared to ambient temperature in a pure aqueous solution, likely because the polymer brushes collapsed at the higher temperature and blocked the binding sites.

Not only brush layers are used for grafting, as Pan et al. [103] also demonstrated the use of a crosslinker (such as methylene bisacrylamide (MBA) to produce a hydrogel layer. This approach widens the scope of this versatile surface-grafting approach. Later on, a new strategy that allows for the more effective controlled synthesis of pure water-compatible MIMs with surface-grafted hydrophilic polymer brushes via facile one-pot RAFTPP was developed, using two types of CTA, a normal RAFT agent and hydrophilic macromolecular chain-transfer agents (Macro-CTAs, such as PEG Macro-CTAs and poly(N-isopropylacrylamide) [PNIPAAm]) [8]. Macro-CTAs play the role of a coCTA and steric stabiliser. The results show a significant increase in both surface hydrophilicity and pure water-compatible template binding properties for grafted MIMs prepared with hydrophilic Macro-CTAs, making this method highly favourable for producing a water-compatible MIM. Another study using Macro-CTAs was conducted by incorporating a fluorescent monomer 2-hydroxyethyl anthrancene-9-carboxylate methacrylate (AnHEMA) into the RAFTPP system [75], giving them analyte binding-induced fluorescence quenching properties. Therefore, the MIMs were able to act as optical chemosensors for direct drug quantification in a biological matrix, skipping any sample pretreatment, thus making it an effective and efficient method.

Considering the great potential of stimuli-responsive MIMs, Ma et al. [71] developed a new MIM that has both thermo- and photoresponsive properties in a pure aqueous environment via RAFTPP by surface-grafting an azo-containing MIM layer and thermo-responsive PNIPAAm brushes. The azo group’s configuration can be controlled by light irradiation, thus generating an alteration in the structural arrangement of the binding sites that affects binding functionalities, resulting in a significant modification of the strength of host-guest interactions. This method resulted in orange-yellow MIP and NIP particles, demonstrating the presence of the azo-containing polymer layer on the core of the MIM. The photoresponsive properties of the grafted MIMs were shown by their photoregulated ability to take up and release the template in pure aqueous solutions. UV light irradiation led to the release of the template from the MIMs into the solution, shown by a decrease in equilibrium template binding capacity. The isomerisation process of the system, when conditioned in the dark, carried the process to template reuptake by the grafted MIMs, shown by an increase in the equilibrium level of template binding capacity. The photoswitching cycle was repeated and the results indicate the reversibility of substrate affinity and binding site configuration by photoswitching the azo chromophores in pure aqueous solutions.

Another study to produce stimuli-responsive MIMs was also conducted by Ma et al. [72], creating MIMs with multiple stimuli (photo, thermo and pH)-responsive properties via surface-initiated RAFTPP of NIPAAm and 2-(dimethylamino)ethyl methacrylate (DMAEMA). Both thermo- and pH-responsive template binding properties proved by the significant decrease in specific template bindings along with rising temperatures from 20 to 50 °C and pH from 6 to 10 at 40 °C. This phenomenon occurs because of higher temperatures and pH most likely, leading the polymer brushes to collapse, making them block the binding sites.

Recent studies has successfully developed RAFTPP for different templates [76,77,78], especially as drug delivery systems [82], different CTA [73,74] and different matrices [104]. As a commonly used approach to produce MIMs, RAFTPP has wide possibilities for further applications in various fields, such as environmental monitoring (e.g., removing toxic compounds from natural products [75]), food analysis, clinical diagnostics, bioimaging and stimuli-responsive drug delivery (see Table 1).

### 3.3. Pickering Emulsion Polymerisation

Pickering emulsion uses dispersed droplets that are stabilised with solid particles, either oil-in-water or water-in-oil (inverse Pickering emulsion). The use of Pickering emulsions must consider the possibility of coalescence between droplets, so stabilising particles are on the surface of droplets [136,137]. The Pickering emulsion method has been used in cosmetics preparation, oil purification and wastewater treatment [138]. The inverse Pickering emulsion is used to produce spherical hydrogel particles [79] (see Table 1). The formation of MIMs requires a template, which is the target compound. Porogens are used as solvents according to template solubility. Commonly used porogens include organic compounds such as toluene [87]; SiO_2_ is important in the Pickering emulsion method as an emulsion stabiliser [50]. Another ingredient that plays a significant role in the formation of fine polymer emulsions is the surfactant. A surfactant that has been used in several studies is Triton-X [48,87].

Yang et al. [87] showed that the mass of SiO_2_ used can affect the diameter of the particles produced. Polymers with 5 mg SiO_2_ resulted in irregular spherical particles, whereas with 15 mg SiO_2_, a regular shape was formed but deemulsification still occurred. The use of more than 30 mg of SiO_2_ resulted in particles with a narrower size distribution, indicating that the particles had a uniform size. The more SiO_2_ added, the smaller the diameter of the particles. Experiments by Binks and Lumbsdon [139] showed that unmodified silica can be used to stabilise oil in water (O/W) emulsions due to the hydrophilicity of Si-OH groups, whereas hydrophobic modified silica can be used to stabilise water in oil (W/O) emulsions. Modified silica can be produced by mixing dried silica and toluene, then adding trimethylsilyl chloride slowly at room temperature. The mixture was evaporated and then stored under argon atmosphere [79]. Besides SiO_2_, lignin can be used as an emulsion stabiliser. However, lignin is hard to eliminate and decompose. However, the exploration of methods utilising lignin has attracted extensive attention [117].

The formation of microsphere polymers using the Pickering emulsion method can produce particles with a more controlled size. This method can also produce either hydrophilic or hydrophobic polymers [138]. The MIM surface is regular, with a uniform pore size distribution and an impressive selective polymer to Malachite Green (MG), despite its lower adsorption capacity as a result of the smaller specific surface area [48].

Varying the porogen volume also contributes to good MIM production. Based on experiments by Yang et al. [87], using different volumes of toluene as the porogen (1.6, 2.6, 3.6, 4.6 and 5.6 mL) resulted in different polymer properties. MIMs with lower and higher porogen volumes had a lower imprinting factor (IF), whereas polymers produced with 3.6 mL as the porogen volume showed the highest IF value. Polymers produced with a porogen volume less than 3.6 mL had fewer accessible recognition sites for the target molecule. A small amount of emulsifier was also needed to avoid demulsification, since the stability of the oil–water phase is affected by the template molecule.

**Figure 5:**
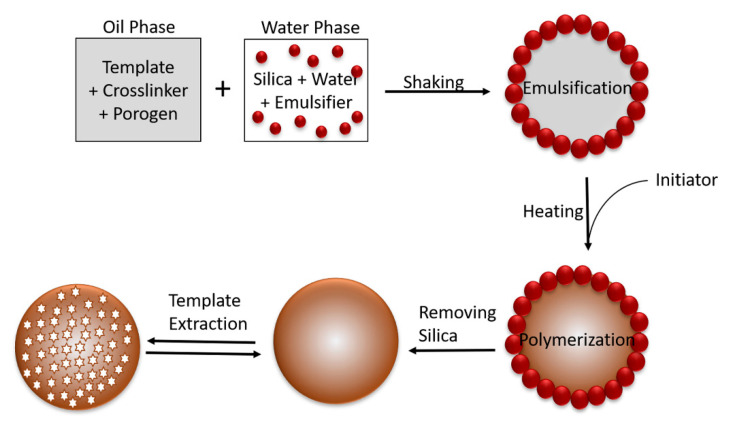
The mechanism of Pickering emulsion polymerisation.

The water phase consisted of the monomer, emulsifier and nanosilica (Figure 5). Sonication treatment was important to disperse SiO_2_ into the water. The oil phase consisted of a crosslinker, template molecules, porogen and initiator as the pore forming agent. Sonication was also performed in this step to homogenise the oil phase mixture. The water and oil phases were then mixed together with agitation. The free radical polymerisation of the monomer was done in a water bath at 70 °C for 16 h triggered by the initiator. The sinking microspheres were cleaned by dipping into acidic solution to remove the silica particles. The last step was template extraction, which was achieved with Soxhlet extraction [48,87,138].

Another method, multi-hollow microspheres, uses the same method as hydrophilic Pickering emulsion. The difference is in the use of Bisphenol A (BPA), as during polymerisation, BPA can trigger multiple nucleation inside the droplet. Hollow MIP spheres encapsulating many sub-spheres were obtained after the BPA was extracted. With accuracy and selective efficiency, these rattle-like spheres also showed remarkably accelerated adsorption kinetics. Comparatively, polymerisation without BPA resulted in hollow spheres with only a single void inside [89].

### 3.4. Suspension Polymerisation

Suspension polymerisation can be used to replace bulk polymerisation with easy operation and avoiding the grinding and sieving steps [118]. MIMs are mostly prepared in organic solvents. It is hard to recognise the template on MIMs in a polar solvent with aqueous suspension polymerisation. Water or a polar solvent can weaken the non-covalent interaction between the monomer and the template.

Based on research by Lai [110], the components needed to make MIMs using the suspension polymerisation method are molecular templates that are drugs or compounds that will be targeted for research, and monomers to be formed as polymers. Several studies have separated drug compounds using methacrylic acid (MAA) as a monomer (see Table 1) [80,120], and non-polar solvents as porogens, crosslinkers and initiators. Substances that play a major role in this method are suspension agents that are mixed with the disperse phase. Certain compounds, such as retinoic acid, were tried by Kim [140], but require catalysts such as triethylamine.

**Figure 6:**
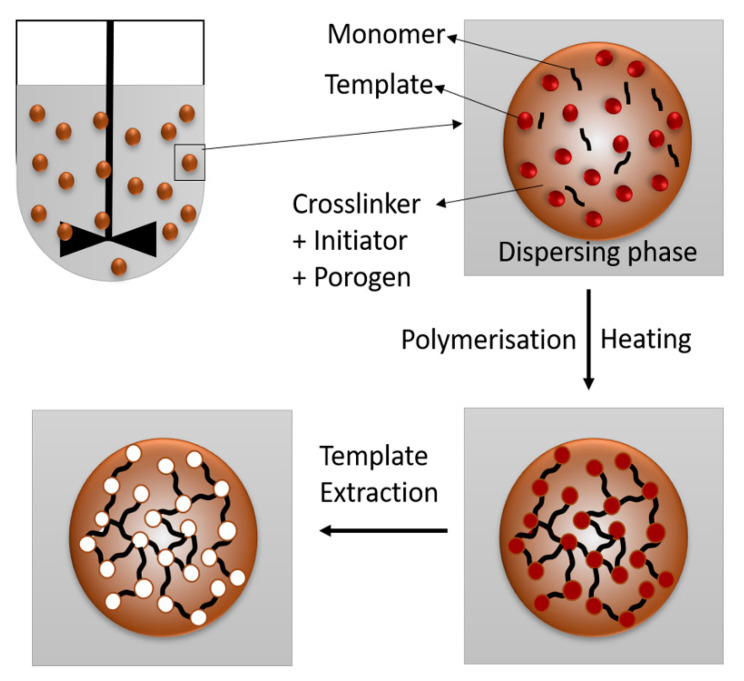
The mechanism of suspension polymerisation.

Suspension polymerisation uses a two-phase system, i.e., a disperse phase and a continuous phase or dispersing phase (Figure 6). The disperse phase contains the template molecule, monomer, crosslinker, initiator and porogen, while the dispersing phase contains water and the dispersing agent. The template molecule and functional monomer are dissolved in the porogen. The crosslinker and the initiator are added to this mixture and sonicated to dissolve. For the dispersing phase, the dispersing agent is dissolved in water at 60 °C under a nitrogen atmosphere in a flanged reactor flask with a mechanical stirrer, reflux condenser, nitrogen inlet and dropping funnel. The mixture has to reach room temperature before being admitted to the flask at 400–600 rpm under a gentle stream (about 60 bubbles per minute) of nitrogen (99.99%). The temperature is then raised to 60–70 °C for the polymerisation process for 24 h. The microspheres have to be washed with a combination of distilled water, methanol and acetic acid [110,120], ethanol [80] or acetone [140].

The ratio between monomers and molecule template must be adjusted—too many or too few monomers will affect the affinity and selectivity of the polymer formed; this was examined by Shi [120] with five different monomer to molecule target ratios of 2:1, 3:1, 4:1, 6:1 and 8:1. The optimum ratio that showed specific binding of chloramphenicol as the molecule template was 4:1, with the highest recovery of 81.6%, while the NIMs with the same ratio were lower at 28.5%. The specific adsorption recovery for each ratio, i.e., 2:1, 3:1, 4:1, 6:1 and 8:1, was 33.5%, 25.9%, 53.1%, 20.1% and 17.6%, respectively.

The reason for the adjustment ratio for the monomer to molecule template has been explained [81]. A lower monomer to template molecule ratio means that the absorption capacity is worse because the insufficient amount of monomers leads to less complexation to the binding site, whereas a higher monomer to template molecule ratio leads to worse imprinting performance, because excessive binding sites cause more non-selective binding with the template molecule.

The effect of the amount of surfactant or suspending agent on the size and distribution of particles has also been assessed. A higher amount of surfactant produces smaller average particle diameters and a narrower size distribution [140]. Gluten and PEG 4000 at 4%, and PVA1788 at 2%, 4% and 6% as a dispersant or suspending agent, were tested by Shi [120]. Different types of dispersants could be seen from the formation of agglomerates. With the use of PVE, a few agglomerates formed, while the use of gluten led to agglomerate blocks, and PEG 4000 formed many agglomerates. Different concentrations of dispersants result in different particle sizes. PVA1788 at 2%, 4% and 8% formed particles with diameters of 120, 80 and 40 µm, respectively. Different dispersing agents such as water and perfluoro-1,3-dimethylcyclohexane were explored by Kim [140] and led to similar structures and sizes, but did not affect the shape significantly.

MIM and NIM polymers have significant differences. Polymers with MIMs have an irregular, rough morphology with many small cavities caused by the template molecule structure. Polymers with NIMs have a more uniform, smooth shape. This shows that MIMs can be a potential sorbent for the separation and enrichment of analytes from a matrix [119]. Geng [80] showed that the IF for erythromycin is 3.86. This indicates that the MIP has high specific adsorption for the template. In other experiments using different template molecules, melamine showed an IF of 4.1, meaning that melamine as a template molecule bound four times higher to MIMs than to NIMs [119].

To sum up the discussion of various polymerisation techniques in the preparation of MIMs, some advantages and disadvantages of each technique are provided in Table 2.

To compare the effects of different polymerisation technique, we made some comparison of binding affinity and IF with the same template molecule. Microsphere polymers made for nicotine with precipitation polymerisation and RAFTPP were made from MAA as a functional monomer [56,57]. Precipitation polymerisation resulted in an IF value of 10.5 for S-nicotine; meanwhile, RAFTPP has IF 3.33 in the synthesis of these two methods using different ratio compositions of template:monomer:crosslinker, namely 1:4:20 for precipitation polymerisation and 1:3:12 for RAFTPP. It could not be concluded whether the lower IF value of RAFTPP made this technique have lower polymer performances due to the different composition of the monomer and crosslinker. The crosslinker in the RAFTPP technique has a lower ratio towards the template and monomer compared to precipitation polymerisation. Liu et al. [141] explained that less crosslinker will create long, free polymer chains within large pore diameters and low mass transfer resistance due to fewer crosslinking sites. Another result from Barros et al. [142] shows that a large amount of functional monomer typically results in more non-specific interaction sites. Meanwhile, inadequate functional groups produce less complexation in the polymerisation process. In other results on imprinted polymers for 17β-estradiol, we found that all polymerisation techniques could give IF more than 1 [40,41,49,50,109]. The highest IF values were obtained from ATRPP techniques with high affinity binding and fast kinetics with 4-VP monomer. Jin Y et al. [52] and Jiang M et al. [88] made bisphenol A microsphere polymer using precipitation polymerisation. A polymer made from 4-VP monomer with 16.7% relative crosslinker to total volume [88] has a higher IF value than 28.6 vol% made by Jin Y [52]. Wei, S et al. [143] reported that the coagulation of the growing polymer microsphere was prevented by the crosslinker, but too large a crosslinker ratio will result in coagulation, because increasing the solvency of the continuous phase swelled the particles’ surfaces and made them more prone to coagulation. Coagulation will lead to unfavourable mass transfer and lower binding. A microsphere-imprinted polymer for bisphenol A made by Pickering emulsion polymerisation has a larger IF value than PP [52,87], but those two methods have lower IF values than ATRPP. These results cannot be used conclude that ATRPP was a better method than the others, since different ratio compositions of template:monomer:crosslinker were used. A summary of three different templates made with different technique of polymerisation can be seen in Table 3. Future investigations to conclude which technique is better still need to be done with the same ratio composition of template:monomer:crosslinker with the same volume and type of solvent.

## 4. Green Aspects in Molecularly Imprinted Microspheres

In developing an economic, reliable, sustainable, and environmentally friendly approach, the reusability and stability of the imprinted material plays an important role. It is well-known that polymer degradation products can also contaminate the sample during its application. There are four main factors that affect the reusability and stability of MIP, namely the crosslinker, crosslinking degree, condition template extraction and functional monomer. These factors were investigated by Kupai et al. [144] using eleven different l-phenylalanine methyl ester (ME)-imprinted polymers in various compositions. Their study conducted an evaluation of long-term stability and reusability via performing adsorption–regeneration cycles up to 100 times. In terms of the crosslinking degree, DVB-based polymers showed a great result where DVB-based polymers can be reused at least 100 times without losing its template affinity under acidic and basic conditions and elevated temperature (65 °C). In contrast with DVB-base polymers, the crosslinking degree of acrylamide and methacrylate-based polymers decreased in both acidic and basic conditions due to their irreversible degradation. As discussed above, both the acrylamide and methacrylate crosslinker are commonly used in MIM preparation. This can be a challenge for future research in developing a long-term stable and reusable formulation of MIMs.

Reducing the use of solvents and energies could also have a significant impact. These aspects were deeply discussed by several studies [145,146,147]. According to these studies, alternative approaches that can be considered include the use of green templates, green monomers, green solvents such as porogens and template removal solvents, green crosslinkers and initiators, energy efficiency, incorporation of ultrasound and microwaves in promoting reaction rates, miniaturized techniques, and the use of computational tools for optimizing both the polymer and synthesis process. Energy efficiency is crucial, since higher energy needs cause significant impacts for the environment, such as global warming. As mentioned above, all CRPP techniques can be carried out at mild temperature conditions, which is beneficial in terms of the environmental aspect.

## 5. Conclusions and Future Perspectives

In this article, several preparation methods to synthesise MIMs and their versatile applications have been discussed. However, we cannot conclude which technique is better because of the absence of studies which conducted with the same ratio composition of template:monomer:crosslinker in the same volume and type of solvent. We believe studies based on improving MIM properties and applications will continue to grow rapidly. Future studies may focus on these following areas.

1. Comparison study. Future investigations to conclude which technique is better still need to be done with the same ratio composition of template:monomer:crosslinker in the same volume and type of solvent.
2. Applicability of MIMs as drug delivery systems. MIMs have excellent potential as drug delivery systems because of their selective binding characteristics and their ability to release the template from the matrix. MIMs can also be used as targeting systems for the recognition of large molecules in gene therapy.
3. Multiple stimuli-responsive MIMs. As there are thermo-, photo-, and pH-responsive MIMs, it is highly possible to develop multiple stimuli-responsive MIMs. Another environmental variable that can be used as a stimulus is biomolecule-responsive, which is the ability to undergo conformational change in response to signal biomolecules.
4. New CRPP methods. Another CRPP could be developed, for example developing nitroxide-mediated precipitation polymerisation (NMPP) by substituting the conventional initiator with a nitroxide compound.
5. The Pickering emulsion method can be an option for developing MIMs with both hydrophobic and hydrophilic properties. A double emulsion Pickering method can be developed for compound separation to achieve a greater degree of selectivity and affinity.
6. Green strategies. Due to the numerous advantages of MIMs and rapid awareness of the importance of green chemistry, challenges such as how to conduct research and developments with a greener approach were provided for researchers to accomplish.

## Figures and Tables

**Table 1:** Applications of molecularly imprinted microspheres (MIMs).

Application	Template	Monomer and Crosslinker	Polymerisation Technique	References
Selective Drug and Metabolite Recognition	17β-estradiol	MAA, EGDMA, TRIM	PP	[40,41]
		4-VP, AM, TRIM	ATRPP	[49]
		MAA, EGDMA	PE	[50]
	Theophylline	MAA, EGDMA, TRIM	PP	[40,41]
		MAA, DVB		[6]
	Caffeine	MAA, EGDMA, TRIM	PP	[40]
	Naproxen, Diclofenac, Toltrazuril	4-VP, MAA, HEMA, MAAm, EGDMA, TRIM, DVB	PP	[51]
	Estradiol (E2)	4-VP, EGDMA	PP	[52]
	Kaempferol	4-VP, EGDMA	PP	[5]
	Terbutylazine	MAA, EGDMA	PP	[53,54]
	Enrofloxacine	MAA, HEMA, DVB, EGDMA, TRIM	PP	[55]
	Nicotine	MAA, TFMAA, DVB	PP	[56]
		MAA, EDGMA	RAFTPP	[57]
	*Trans*-aconitic acid	MAA, TRIM	PP	[58]
	l-2-chloromandelic acid	AM, BDDA	PP	[59]
	Cinchonidine	MAA, HEMA, DVB	PP	[60]
	Morphine	MAA, TRIM	PP	[61]
	Cinnamic Acid	AA, DVB	PP	[62]
	Piperine	AA, EGDMA	PP	[63]
	Mannose-tryptophan	MMA, DVB	PP	[64]
	2,4-diamino-6-methyl-1,3,5-triazine, cyromazine, trimethoprim	MAA, DVB	PP	[65]
	Sulfamethazine	4-VP, HEMA, EGDMA	ATRPP	[66]
	Glutathione	4-VP, EGDMA	ILRPP	[67]
	Thymopentin	p-CMS, VI, EGDMA	ILRPP	[68]
	Vanillin	MAA, EDGMA	RAFTPP	[69]
	Propranolol	MAA, DVB	PP	[70]
		MPABA, NIPAAm, EGDMA	RAFTPP	[71]
		4-VP, NIPAAm, MPABA, DMAEMA, EGDMA		[72]
		MAA, EGDMA		[73]
		MAA, HEMA, EGDMA		[74]
	Tetracycline	MAA, HEMA, AnHEMA, EGDMA	RAFTPP	[75]
		MAA, EGDMA		[76]
	Quercetin	2-VP, EGDMA	RAFTPP	[77]
	Aristolochic Acid I	AA, EGDMA	RAFTPP	[78]
	Isopropylaminopropanediol	MAA, EGDMA, AIBN	PE	[79]
	Erythromycin	MAA, EGDMA	SP	[80]
	YPLG	MAA, EGDMA	SP	[81]
Controlled Drug Release	Paclitaxel	MAA, EGDMA	RAFTPP	[82]
	Adhenosine 5′-monophosphate	DMAEM, NIPAAm, MBAM	PE	[79]
	Vancomycin	HEMA, DEAEMA	PP	[83]
	Sunitib	MAA	PP	[84]
	1,4-dimethyl-6-hydroxy-9*H*-carbazole (CAB1)	MAA, EGDMA	PP	[85]
	1-(1-naphthyl)ethylamine	Macrocyclic	SP	[86]
Environmental Contaminants	Tebuconazole	4-VP, EGDMA	PP	[52]
	Bisphenol A	4-VP, EGDMA	PP	[52]
			ATRPP	[1]
			PE	[87]
		4-VP, MAA, EGDMA, TRIM	PP	[88]
		4-VP, DVB	PE	[89]
	Monocrotophos	MAA, EGDMA	PP	[90]
	Difenoconazole	HPMA, EGDMA	PP	[91]
	Azoxystrobin	HPMA, EGDMA	PP	[92]
	Carbaryl	MAA, EGDMA	PP	[93]
	Di(2-ethylhexyl)phthalate	MAA, EGDMA, TRIM	PP	[94]
	Simetryne	ABA, DVB	PP	[95]
	Cyhalothrin	AM, EGDMA	PP	[96]
	Polystyrene	MAA, DVB	PP	[97]
	*p*-nitroaniline	IL, EGDMA	PP	[98]
	Diclofenac	2-VP, EGDMA	PP	[99]
	2,4-Dichlorophenoxy- acetic acid	MAzoPy, EGDMA	ATRPP	[100]
		4-VP, NIPAAm, EGDMA	ILRPP	[101]
		4-VP, EGDMA	RAFTPP	[5,8]
		4-VP, NIPAAm, EGDMA		[102]
		4-VP, HEMA, EGDMA		[103,104]
	Pyrazosulfuron- ethyl	MAA, 4-VP, AA, EGDMA, DVB	RAFTPP	[105]
		MAA, EGDMA		[106]
Sensor	Dipyridamole	MAA, EGDMA	PP	[107]
	Enrofloxacine	MAA, HEMA, DVB, EGDMA	PP	[108]
MISPE	17β-estradiol	4-VP, EGDMA	PP	[109]
	4-aminopyridine	MAA, EGDMA	SP	[110]
Extraction From Natural Ingredients or Food	Curcumin	4-VP, MAA, MAM, DVB	PP	[111]
	Dimethoate	MAA, MMA, AM, EGDMA	PP	[112]
	Gallic Acid	AA, EGDMA	PP	[113]
	Caffeic Acid	4-VP, DVB	PP	[114]
	Glutathione	MAA, DVB	PP	[115]
	Atrazine	MAA, EGDMA	RAFTPP	[116]
	Matrine	MAA, EGDMA	PE	[117]
	Melamine	MAA, EGDMA	SP	[118,119]
	Chloramphenicol	DEAEM, EGDMA	SP	[120]

PP, Precipitation Polymerisation; ATRPP, Atom Transfer Radical Precipitation Polymerisation; ILRPP, Iniferter-induced ‘Living’ Radical Precipitation Polymerisation; RAFTPP, Reversible Addition-Fragmentation chain Transfer Precipitation Polymerisation; PE, Pickering Emulsion polymerisation; SP, Suspension Polymerisation; MAA, methacrylic acid; HEMA, 2-hydroxyethyl methacrylate; DVB, divinylbenzene; EGDMA, ethylene glycol dimethacrylate; TRIM, trimethylolpropane trimethacrylate; TFMAA, 2-(trifluoromethyl)acrylic acid; HPMA, 2-hydroxypropyl methacrylate; AM, acrylamide; BDDA, 1,4-butanediyl diacrylate; 4-VP, 4-vinylpyridine; MAM, methacrylamide; MMA, methyl methacrylate; ABA, allobarbital; AA, acryic acid; IL, 3-(anthracen-9-ylmethyl)-1-vinyl-1H-imidazol-3-ium chloride; 2-VP, 2-vinylpyridine; MAAm, methacrylamide; MAzoPy, 4-((4-methacryloyloxy)phenylazo)pyridine; p-CMS, 4-(chloromethyl) styrene; VI, 1-vinylimidazole; MPABA, 4-((4-Methacryloyloxy)phenylazo) benzoic acid; DMAEM, 2-(dimethylamino)ethyl methacrylate; AnHEMA, (2-hydro-xyethyl anthrancene-9-carboxylate) methacrylate; DEAEM, 2-(diethylamino) ethyl methacrylate; NIPAAm, N-isopropylacrylamide; MBAM, N,N′-Methylenebis(acrylamide); YPLG, Tyr–Pro–Leu–Gly–NH.

**Table 2:** Advantages and disadvantages of different polymerisation technique in preparation of molecularly imprinted microspheres.

Polymerisation Technique	Advantages	Diadvantages
Precipitation Polymerisation	Easy to operate. Requires no stabiliser or surfactant. Good control of particle sizes and morphology.	Commonly using high amount of solvent.^1^ Low control of polymerisation rate. Commonly do not possess ‘living’ groups on the polymer surface.
Controlled/‘living’ radical precipitation polymerisation (CRPP) ^2^	Requires no stabiliser or surfactant. High control of polymerisation rate, composition, and molecular weight. Able to do advanced surface modification because the presents of ‘living’ groups on the polymer surface. Can performed under mild reaction conditions	
Atom Transfer Radical Precipitation Polymerisation (ATRPP)	Wide scope of monomer, initiator and catalyst utilization. The obtained MIMs are generally end-capped with a reactive halogen groups. Relatively low-cost.	May has limited application due to the use of large amounts of acidic functional monomers or templates that could possibly deactivate the metal catalyst
Iniferter-induced ‘Living’ Radical Precipitation Polymerisation (ILRPP)	Compatible with many molecular imprinting systems The obtained MIMs are generally end-capped with an iniferter groups.	Less controlled compared to ATRPP and RAFTPP
Reversible Addition-Fragmentation Chain Transfer Precipitation Polymerisation (RAFTPP)	Wide scope of monomers (nearly all monomers) The obtained MIMs are generally end-capped with a dithioester groups. Well suited for the preparation of high molecular weight polymers.	The presence of dithioester groups makes the obtained MIMs coloured dan may have some odours for low molar mass species that might require radical chemistry for removal and displacement. The presence of a continuously generated new short chains which terminate faster than the longer chains.
Pickering Emulsion	The addition of BPA can produce multi-hollow microsphere Require use of surfactants	Have more possibility to coalescence between the droplets Concentrations of solid phase must be considered to avoid coalescence
Suspension Polymerisation	Dispersing agent must be inert with the template, monomer, crosslinker and initiator Required use of special dispersing phases	The template on MIMs in a polar solvent with aqueous suspension polymerisation have weaker covalent interactions with the monomer.

^1^ Can be overcome using modified precipitation polymerisation (MPP); ^2^ Applied to ATRPP, ILRPP and RAFTPP.

**Table 3:** Comparation of different polymerisation in preparation of molecularly imprinted microspheres.

Template	Monomer	PP		ATRPP		RAFTPP		PE	
		Binding Affinity	IF	Binding Affinity	IF	Binding Affinity	IF	Binding Affinity	IF
17β-estradiol	MAA	More than 50% analyte [40,41]	2.3 [40] 4.0 [41]	-	-	-	-	30–60% analyte [50]	3.0 [50]
	4-VP	0.75 mg/g [109]	4.55 [109]	180.65 mg/g [49]	6.67–7.38 [49]	-	-	-	-
Nicotine	MAA	Not mention in the article [56]	10.5 [56]	-	-	Not mention in the article	3.33 [57]	-	-
Bisphenol A	4-VP	Not mention in the article	3.91 [52] 4.83 [88]	Not mention in the article	10 [1]	-	-	1.32 mg/g [87]	6.5 [87]

PP, precipitation polymerisation; ATRPP, atom transfer radical precipitation polymerisation; RAFTPP, reversible addition-fragmentation chain transfer precipitation polymerisation; PE, Pickering emulsion polymerisation; IF, imprinting factor; MAA, methacrylic acid; 4-VP, 4-vinylpyridine; -, no resource found in related technique.
